# Understanding Trust Determinants in a Live Chat Service on Familial Cancer: Qualitative Triangulation Study With Focus Groups and Interviews in Germany

**DOI:** 10.2196/44707

**Published:** 2023-08-23

**Authors:** Hanna Luetke Lanfer, Doreen Reifegerste, Annika Berg, Paula Memenga, Eva Baumann, Winja Weber, Julia Geulen, Anne Müller, Andrea Hahne, Susanne Weg-Remers

**Affiliations:** 1 School of Public Health Bielefeld University Bielefeld Germany; 2 Department of Journalism and Communication Research Hochschule für Musik, Theater und Medien Hannover Hannover Germany; 3 Krebsinformationsdienst Heidelberg Germany; 4 BRCA-Netzwerk Bonn Germany

**Keywords:** trust, live chat, web-based health seeking, qualitative research, cancer

## Abstract

**Background:**

In dealing with familial cancer risk, seeking web-based health information can be a coping strategy for different stakeholder groups (ie, patients, relatives, and those suspecting an elevated familial cancer risk). In the vast digital landscape marked by a varied quality of web-based information and evolving technologies, trust emerges as a pivotal factor, guiding the process of health information seeking and interacting with digital health services. This trust formation in health information can be conceptualized as context dependent and multidimensional, involving 3 key dimensions: information seeker (trustor), information provider (trustee), and medium or platform (application). Owing to the rapid changes in the digital context, it is critical to understand how seekers form trust in new services, given the interplay among these different dimensions. An example of such a new service is a live chat operated by physicians for the general public with personalized cancer-related information and a focus on familial cancer risk.

**Objective:**

To gain a comprehensive picture of trust formation in a cancer-related live chat service, this study investigates the 3 dimensions of trust—trustor, trustee, and application—and their respective relevant characteristics based on a model of trust in web-based health information. In addition, the study aims to compare these characteristics across the 3 different stakeholder groups, with the goal to enhance the service’s trustworthiness for each group.

**Methods:**

This qualitative study triangulated the different perspectives of medical cancer advisers, advisers from cancer support groups, and members of the public in interviews and focus group discussions to explore the 3 dimensions of trust—trustor, trustee, and application—and their determinants for a new live chat service for familial cancer risk to be implemented at the German Cancer Information Service.

**Results:**

The results indicate that experience with familial cancer risk is the key trustor characteristic to using, and trusting information provided by, the live chat service. The live chat might also be particularly valuable for people from minority groups who have unmet needs from physician-patient interactions. Participants highlighted trustee characteristics such as ability, benevolence, integrity, and humanness (ie, not a chatbot) as pivotal in a trustworthy cancer live chat service. Application-related characteristics, including the reputation of the institution, user-centric design, modern technology, and visual appeal, were also deemed essential. Despite the different backgrounds and sociodemographics of the 3 stakeholder groups, many overlaps were found among the 3 trust dimensions and their respective characteristics.

**Conclusions:**

Trust in a live chat for cancer information is formed by different dimensions and characteristics of trust. This study underscores the importance of understanding trust formation in digital health services and suggests potential enhancements for effective, trustworthy interactions in live chat services (eg, by providing biographies of the human medical experts to differentiate them from artificial intelligence chatbots).

## Introduction

### Trust in Health Information Seeking

Cancer incidence in some families surpasses what might be expected by chance, indicating a possible familial risk of cancer. This risk can be tied to shared lifestyle factors (eg, unhealthy dietary habits), common environmental factors (eg, radiation exposure), or a hereditary predisposition, among others [1,2]. A suspected or confirmed familial cancer risk often results in fear and uncertainty for the affected individuals and their families [3].

This fear and uncertainty, coupled with the critical health implications of familial cancer risk, intensify the necessity for accurate, reliable information. Web-based health information becomes a crucial coping strategy for those facing familial cancer risk, including patients, relatives, and those suspecting an elevated risk [3]. However, the quality of web-based cancer information varies extensively, making trust a pivotal factor in health information seeking [4].

Trust refers to accepting one’s vulnerability and relying on the action of another party, with no assurance regarding how the other party will behave [5-8]. In the intricate nexus of patient-provider relationships, trust and power are inherently linked—trust cannot be conceived without power because it always goes hand in hand with uncertainty. This delicate balance of trust, power, and information has profound implications for health care experiences and outcomes [9]. In the traditional health care setting, physicians, as the primary sources of medical information, held a significant power position born from the knowledge asymmetry, high vulnerability, and dependence that inherently characterize patient-provider interactions. This asymmetry is compounded by the complexities and inherent uncertainties of medical evidence, often long term, inconsistent, contradictory, and challenging to access and understand [10,11].

The advent of the digital age has started reshaping this asymmetry because patients increasingly resort to the internet for health-related information, entering health care interactions with pregained knowledge [9,12]. On the one hand, this shift empowers patients and challenges the traditional power asymmetry in health care. On the other hand, it introduces new uncertainties because the information that patients find on the internet may not always be accurate or reliable [13,14].

With the advent and continual evolution of digital applications, the landscape of trust in health information has been substantially transformed [15,16]. First, digital platforms have amplified the accessibility of information from diverse sources. Although formal (eg, government institutions, health care providers, and universities), commercial (eg, health care–related companies), and informal (content generated by laypeople and social media) sources have always existed, the digital era has blurred the lines among these categories [17,18]. The relative ease of web-based publishing can lead to wide variability in the quality of information, the degree of scientific evidence supporting it, and its understandability for laypeople [19]. Moreover, in the predigital era, gatekeepers of information, such as publishers or editorial boards, served as initial filters, whereas these traditional gatekeepers are less present or visible in digital spheres, which results in a more complex environment for trust formation [20,21]. Studies show that the public consults fellow laypeople for health information, including friends, family, and even strangers on social media, as well as automated feedback on tracking apps [22,23]. Such interactions are an indication of trust, whereby the same individuals may mistrust evidence-based information from the government or health experts. This dichotomy was notably exemplified during the COVID-19 pandemic [22,24-26].

Second, users are not only deciding whom to trust for web-based health information but also how to engage with it, given the diverse array of information providers and service formats available [27,28]. The interaction with health information is no longer a 1-way street; users can now engage with it either passively, by consuming static content such as text or video, or actively, by participating in interpersonal or group settings with both human and automated entities (eg, in live chats) [29,30].

This changing landscape of information access places new communication challenges on patients’ (offline) conversations with clinical physicians, for whom it might be overwhelming to navigate conversations with preinformed patients, address potential misinformation, and do so within the constraints of limited consultation time [31]. Given the vast disparities in the quality of web-based cancer information, the role of trust becomes paramount in the process of providing and seeking health information, intertwined with the selection of information source, the choice of platform, and the influence of social network endorsements. This dynamic interplay of trust determines how, where, and from whom individuals seek health information [32,33]. It is therefore critical to continually re-evaluate and understand how users form trust in new services, given the intricate interplay among the information seeker, attributes of the information, and its source [19,34]. An example of such a new service is a live chat offered by the German Cancer Information Service (CIS), a government-funded service that provides individual evidence-based cancer information to the public [35].

By live chat, we refer to a text-based real-time chat service for interactions between medical experts from the German CIS and internet users to be implemented on a new CIS subsite. The live chat will have a topical focus on familial cancer, that is, cancer caused by predisposing germline mutations in certain genes as well as cancer caused by an unhealthy lifestyle or environmental factors shared by family members [1,36]. The focus will be primarily on patients at risk for familial cancer and their immediate networks. Given that germline mutations tend to affect younger patients more than other forms of cancer [36], the live chat has been selected as an appropriate tool, in line with the communication preferences of younger generations [21,34].

The live chat will combine the characteristics of static internet content and governmental sources with interactions between laypeople and medical experts. Using the chat service, inquirers will benefit from easy personalized access to evidence-based information provided by physicians—patients’ most trusted and preferred source of health information [37]—but independent from their personal medical provider (eg, their general physician or oncologist). In addressing familial cancer, the live chat service aims to bridge the gap between the heightened need for trustworthy information and the uncertainty faced by those at (potential) increased familial cancer risk. By providing reliable evidence-based information in an accessible interactive format, the service seeks to support and empower individuals to deal with this risk and associated concerns.

### Conceptual Background

#### Live Chats

In recent years, real-time live chats have become standard in many digital commercial customer services (eg, to answer questions, place orders, and handle complaints). These have distinctive features compared with static website information or user-generated information in web-based forums, on social media, or asynchronous exchange over email. These services allow inquirers to maintain a high degree of anonymity, receive synchronous personalized feedback or support from a selected service, control the pace of the conversation, and gain access to the service irrespective of where they are (eg, not requiring a quiet surrounding as would be needed for a telephone call) [38,39]. They are particularly popular among younger digitally oriented web-based users [40]. Live chats can be operated by humans or digital conversational agents, otherwise known as automated chatbots, or a combination of both [40].

Because of their popularity and their unique features, live chats are also increasingly used in health-related settings, especially in crisis or acute-risk situations (eg, abuse, mental health and suicide, and addiction support; for a systematic review, refer to the study by Brody et al [38]). Despite the frequent use of live chats, challenges in forming trust between the inquirer and the respondent in a live chat have been noted, especially when it comes to handling more complex requests [41-43]. Despite this, the exploration of trust in live chat encounters has received relatively little scholarly attention. Although trust in conversational agents has been an important area of research [43,44], including traditional communication methods such as telephone conversations [45], there is still a notable knowledge gap when it comes to complex topics such as cancer. Given the unique complexities that cancer-related questions introduce to the chat interaction, it is essential to understand the specific factors that foster trust in this service. This need is further underscored by the continual emergence of new applications, each necessitating an examination of trust dynamics.

#### Conceptualizing Trust

##### Overview

Despite trust being a well-researched area of study, its definition and conceptualization remain contested [32,46]. One frequently quoted definition of trust is “the willingness of a party to be vulnerable to the actions of another party based on the expectation that the other will perform a particular action important to the trustor, irrespective of the ability to monitor or control that other party” [7]. When comparing various concepts of trust, there are 3 aspects that are essential.

First, trust is necessary for situations of risk or uncertainty when a person, *the trustor*, cannot control the outcomes of the action of another party or entity, *the trustee*. Trust then acts as a mechanism to accept a risk and one’s vulnerability in the hope that the trustee has positive intentions [4,7,46,47]. Hence, trust is deemed essential in health information and support seeking, given the trustor’s dual uncertainties: both as a person facing the risk of disease and its unknown outcomes and as a person dependent on another party or entity for the provision of trustworthy information. This is especially relevant for information regarding cancer, where the risk of severe health consequences is considered very great (often regarded as deadly in public opinion), because of the information variability (owing to intensive ever-developing research) [48,49].

Second, trust was originally conceptualized as an interpersonal concept, where the trustee was a human (eg, a physician) [7]. It has since been extended to other entities such as institutions (eg, the health care sector) or toward machines (eg, diagnostic programs in health care). In face-to-face encounters, trustors have been shown to rely on heuristics (eg, voice, gesture, or clothes) to draw conclusions about the trustee’s trustworthiness [50-52]. However, trust can also develop interpersonally while being mediated or enabled by digital applications (eg, via a live chat). Here, users interact with a technological interface that often lacks the human and social cues of a face-to-face encounter. In these cases, the features of the technology play a role in trust formation because users also rely on *application-related determinants* (eg, usability-related feature forms such as text length adapted to the medium and the use of pictures) [4,41,43]. Ergo, trust becomes a triangular mechanism here influenced by the determinants of the trustor, the trustee, and the application [32,43].

Third, trust is a human’s psychological state based on emotional and cognitive components [7,47]. In this respect, trust does not miraculously appear but is subjective, founded on several determinants, and develops over time [53,54].

**Figure 1:**
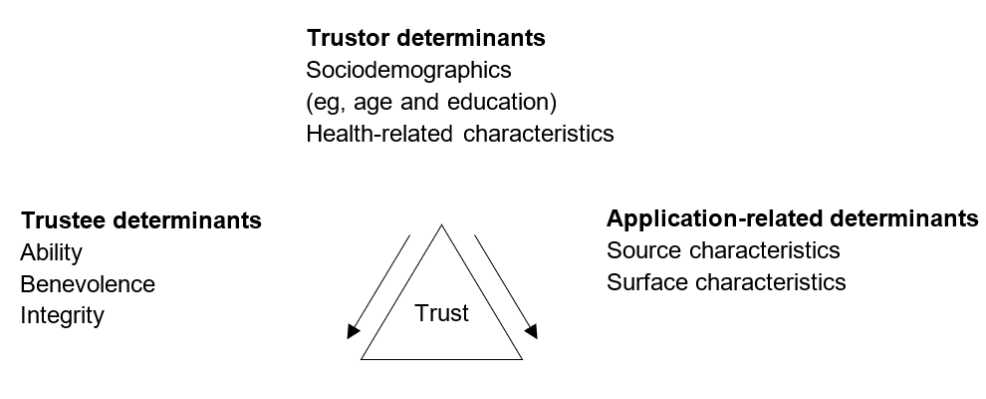
The 3 dimensions of trust and their determinants for web-based health information (adapted from Lucassen and Schraagen [55] and Mayer et al [7]).

Thus, trust is a multidimensional concept that includes the following three dimensions [7,8], which are based on a triangular concept for trust in web-based health information (Figure 1): (1) the *trustor*, who intends to trust; (2) the *trustee*, whom the trustor intends to trust and therefore evaluates; and (3) the *application*, which the trustor similarly intends to trust and evaluates. The formation of trust is influenced by (dimension-specific) determinants for each of these 3 dimensions.

##### Trustor Determinants

Various studies have explored which determinants of the trustor promote trust when navigating the internet for web-based health information and distinguishing between their perceptions of trustworthy sources and those of less credible sources. A 2017 systematic review [56] of 24 articles showed that among a range of *sociodemographic factors*, age was a key determinant influencing source selection and trust perceptions in web-based health information. Older adults had overall the lowest trust in web-based resources and experienced difficulties in navigating and evaluating the quality of health information. A similar lack of evaluation skills—but accompanied here by overall high trust levels in web-based information—was found at the opposite end of the age spectrum. This was especially so among teenagers and young adults who primarily based their assessment of information on superficial cues such as aesthetics and ease of use. The intermediate age group (persons aged 25-55 years) was overall characterized as the most critical with regard to content in their evaluation of health information, while showing a greater level of trust in web-based information than the group of older adults. In this intermediate age group, education and income level were influential demographic factors, whereas in the other age groups, these factors were weakly associated and had minimal influence on the outcomes. Higher educational levels and higher incomes among middle-aged internet users were associated with more discernment when judging health information and an increased willingness to use and trust the selected web-based health information [56]. Although several studies have shown that generally women search more often for health information on the internet [57,58], the studies under review did not provide distinct insights into the intersections of other sociodemographic characteristics in relation to gender and trust in web-based health information [56]. In addition, other reviews and studies have provided evidence that experience with a certain health condition (eg, cancer [4]) as well as digital literacy and familiarity in using and navigating web-based tools [15,59,60] were associated with more frequent web-based health information seeking and judgmental skills regarding the trustworthiness of different sources.

##### Trustee Determinants

From their literature review, Mayer et al [7] identified 3 reoccurring interrelated determinants of the trustee that contribute to their trustworthiness by the trustor. The 3 determinants, which continue to be applied in current studies and theories of trust, including in web-based environments [61,62], are as follows: *ability* refers to specific skills and competencies that grant the trustee trust to perform a certain task, whereas they might not be deemed trustworthy in a different area [7]; *benevolence* describes the extent to which the trustee is believed to act upon the best interests and well-being of the trustor; and *integrity* involves some perceived principles or values of the trustee (eg, honesty and discretion) that the trustor deems acceptable.

##### Application-Related Determinants

Apart from the interpersonal relationship between trustor and trustee, trust can also be displayed toward the characteristics of an application through which the information is shared [4,55]. These determinants are particularly relevant in the web-based context where the appearance of a website or application is generally the first impression a user gets before navigating to any other application (eg, a chat service) [43]. In their information trust model, Lucassen and Schraagen [55] propose a distinction between source determinants and surface determinants. *Source* determinants refer to how the trustor assesses the design and appearance of a website or application to judge an organization’s reputation and authority in the domain in which it operates. *Surface* determinants include the trustor’s assessment of the communicative features of the application, including text length, the use of pictures, and the communication itself.

In sum, the literature shows that trust in health information is a multidimensional construct, influenced by determinants on (at least) 3 dimensions and related determinants for each dimension. For trust formation in web-based health information (where the trustor-trustee encounter is mediated by technology), the trustee and application-related determinants are shaped by the trustor and their expectation of relevant characteristics. For an example of a live chat for familial cancer with these 3 dimensions of trust and their respective determinants conceptualized in a triangular model, refer to Figure 1.

As trust is subjective and based on a trustor’s perception and assessment, trust can be enhanced if it aligns with the trustor’s expectations of trustee- and application-related characteristics. Hence, to develop a trustworthy live chat to answer questions regarding familial cancer at the German CIS, we aimed to investigate the determinants for each dimension from the perspective of the trustor based on this model for the live chat service. First, to carry this out from the perspective of the trustor, we posed the following research questions (RQs):

- RQ1: What are the relevant determinants for the 3 trust dimensions for the CIS live chat?
  RQ1a: What are the relevant determinants of the trustor?
  RQ1b: What are the relevant trustee-related determinants that influence a trustor’s trust?
  RQ1c: What are the relevant application-related characteristics that influence a trustor’s trust?

Second, to gain a comprehensive picture of trust formation in a cancer-related live chat service, we explored these determinants from the perspective of the trustee, that is, employees of the CIS who currently operate the institution’s telephone and email service and might work in the live chat team in the future. Third, we brought together the perspectives of trustor and trustees and looked at volunteers from different cancer support groups (CSGs; persons who have been patients with cancer as well as counseled others). This study focused on persons with familial cancer and their families, that is, persons at risk owing to a germline mutation in a cancer-predisposing gene, because, in these families, the need for individual trustworthy information is particularly great [63,64].

To integrate the perspectives of these 3 distinct stakeholders in our analysis, we aimed to further explore differences regarding certain perceived determinants by posing the following RQ:

- RQ2: How do the determinants of trust vary across different stakeholder perspectives?

## Methods

### Study Design

This qualitative study with focus groups and interviews is part of a larger collaborative project among 2 German universities, the German CIS, and the BRCA Network, a CSG for familial cancer. This project seeks to combine the design and implementation of a live chat service focusing on familial cancer at the German CIS with formative and summative qualitative and quantitative evaluations. The exploratory study presented in this paper was the first study—a formative qualitative evaluation—to be carried out within the project at an early stage in the development process of the live chat service.

### Participants and Procedures

#### Overview

To gain a comprehensive picture of trust formation in preparation for implementing a cancer-related live chat service, three sets of stakeholders with distinctive perspectives and expertise were recruited (Tables 1 and 2): (1) medical cancer advisers at the CIS (for interviews), (2) volunteers from different CSGs (for interviews), and (3) digitally oriented members of the public, including people with a known genetic mutation for familial cancer and others with none, who could be potential users of the live chat service (for 7 focus group discussions).

**Table 1:** Organizational background of interview participants (n=16).

Characteristic			Participants, n (%)
**Organizational background**			
	Cancer Information Service	7 (44)	
	Cancer support groups	9 (56)	
**Sex**			
	Female	13 (81)	
	Male	3 (19)	

**Table 2:** Sociodemographic data and health-related characteristics of focus group participants (n=42).

Characteristic		Participants, n (%)
**Group**		
	Mutation carrier with a cancer diagnosis (3 groups)	20 (48)
	Mutation carrier (2 groups)	11 (26)
	General public (2 groups)	11 (26)
**Sex**		
	Female	28 (67)
	Male	14 (33)
**Age (years)**		
	18-29	13 (31)
	30-39	17 (41)
	40-49	6 (14)
	≥50	6 (14)
**Education**		
	Academic degree	27 (64)
	Nonacademic degree	15 (36)

#### Medical Cancer Advisers

Medical cancer advisers who are currently working in the organization’s research, communication, email, or telephone services and who might become future respondents of the live chat were recruited based on the suggestions of the head of the CIS [35]. Interviews were conducted via the videoconference tool *Zoom* (Zoom Video Communications, Inc) in September-October 2021. The interview participants in this sample did not receive an incentive for participation.

#### Volunteers

Volunteers from different CSGs across Germany were identified based on suggestions from our collaborative partner, the BRCA Network [65]. Representatives from CSGs who serve patients with familial cancer risk were included in the sample for the dual role as (former) patients with cancer and as advisers to other patients. The interview procedures followed the same principles as those described for the medical advisers.

#### Potential Users

Potential users were recruited to participate in focus group discussions. They were divided into three different categories: participants with a known genetic mutation (mutation carriers) (1) with cancer experience and (2) without cancer experience, as well as (3) members of the public without a known genetic mutation and a general interest in cancer prevention and digital health.

After providing informed consent, a link to a short web-based survey with questions about their history with genetic cancer, interest and experience in digital health, and sociodemographic data was sent to each person to determine whether they were suitable for inclusion in the sample. For those who participated in the survey and fit 1 of the 3 categories, focus groups were held in October-November 2021 via Zoom. Each focus group participant received €25 (US $28.25) as compensation for participation. All interviews and focus groups were led by the first author, who has extensive experience in conducting interviews and group discussions, with support from a research assistant. Data were collected in German, and the quotations cited in this paper are translated versions.

### Interview and Focus Group Discussion Protocols

Semistructured interview and focus group discussion protocols were developed in line with the RQs. The interview participants were asked about prior experiences, difficulties, and demands with regard to advising persons who had questions about familial cancer. We asked questions about their professional experience with live chat services and different requirements for the creation of a trustworthy service (related to the trustee and the application).

The focus group participants were asked about their experiences as health service users as well as their strategies and selection criteria in searching for web-based health information. Other questions referred to (trust) experiences with live chat services in general, requirements for a trustworthy cancer live chat application, and the responding trustee.

### Data Analysis

All interviews and focus group discussions were transcribed verbatim. Interview and focus group data were initially analyzed separately following the procedures of qualitative content analysis [66]: an initial coding frame was built in line with the previously described themes of the 2 protocols (deductive procedure), and new subcategories were generated as derived from the data (inductive procedure). This step was followed by axial coding in orientation to grounded theory [67], as subcategories were reviewed, linked, aggregated, and defined to ensure that they were mutually exclusive. All transcripts, coded segments, and finalized coding frames were read once more carefully before comparing the 2 data sets. Coded transcripts were read again to determine whether themes and subcategories were suited to be matched. Data were coded by the first author and a research assistant. Qualitative data analysis was computer assisted: we used MAXQDA software (VERBI GmbH). Three of the data sets with deductive coding (2 interviews and 1 focus group discussion) were coded by 2 coders to determine intercoder reliability. Intercoder reliability and percentage agreement were assessed using ReCal [68]. Agreement between the coders was 86% (Cohen κ=0.8), which suggests substantial agreement [69]. Disagreements in coding occurred mostly in the area of communication requirements, which led to further refinements of these codes.

### Ethics Approval

The study received ethics approval from Bielefeld University (2021-186).

### Informed Consent

The participants were emailed an information sheet and consent form that explained the benefits and risks of taking part in the study and provided detailed information on data protection and confidentiality. Participants were only invited to one of the focus groups or interview appointments once they had signed and returned the consent form.

## Results

### Overview

After a brief description of the demographic data of our participants, the *Results* subsections outline the 3 dimensions—trustor, trustee, and application—and their trust determinants in response to RQ1. Study participants described a range of features and characteristics in relation to our 3 dimensions that they considered important for the operation of a live chat service with a focus on (familial) cancer. It is important to note that not all features were mentioned in relation to the service’s trustworthiness (eg, having service hours that suited working people). Nonetheless, it seems that the overall user centeredness of the service contributes to the overall perceived trustworthiness of the chat service offer.

In the following subsections, we present a comparison of the 3 samples in response to RQ2. Participants are distinguished by their organizational background (CIS or CSG). Focus group participants are referred to by their category: *mutation carrier with cancer diagnosis*, *mutation carrier* (person with a diagnosed gene mutation but no cancer diagnosis), and *general public* (person with neither a known genetic mutation nor cancer). In addition, each participant’s sex and, in the case of focus group participants, age are stated.

### Sample Description

The sample in this study included 7 interviewees from the CIS, 9 interviewees from CSGs (Table 1), and 42 participants in 7 focus group discussions who represented the potential users of the future live chat service (Table 2). Of the total 58 participants in this study, 41 (71%) were women. Of the 42 focus group participants, 27 (64%) had a university degree, and 30 (71%) were aged <40 years.

### Relevant Determinants for a Live Chat Service (RQ1)

#### Overview

This subsection outlines the determinants of trust for a live chat service divided into three dimensions: (1) trustor-related determinants, (2) trustee-related determinants, and (3) application-related determinants. A summary of our coding scheme with subcategories for each dimension derived from theory and data can be found in Table 3.

**Table 3:** Determinants of trust in subcategories as derived from theory and data.

Dimensions and subcategories from theory		Subcategories from data
**Trustee**		
	Sociodemographics	Gender identity Age Education Member of a minority group (eg, the LGBTQIA+^a^ community)
	Health-related characteristics	Personal experience with predisposing gene mutations
**Trustor**		
	Not applicable	Humanness
	Ability	Professional (medical) expertise Communication skills
	Benevolence	Empathy Neutrality
	Integrity	Honesty Discretion
**Application**		
	Source features	Institutional reputation of the website and application Appearance of the website and application User centeredness of the website and application Use of state-of-the-art technology
	Surface features	Possibility of remaining anonymous Change of medium Fast dialogic communication

^a^LGBTQIA+: lesbian, gay, bisexual, transgender, queer, intersex, asexual, and similar minority.

#### Relevant Determinants of the Trustor

Participants from the interview and focus group samples agreed that the most relevant factor for trusting a live chat for information on familial cancer was a personal need, that is, a *personal experience with predisposing gene mutations*, as a patient who had received a cancer diagnosis, a relative who might be affected, or a partner or close friend asking for information on behalf of a patient. Although patients receive a considerable amount of information from their attending physicians, suspected cases (eg, relatives and friends) do not have the same opportunities to speak with medical personnel and thus have a greater need for reliable information. A woman who at the time of data collection had been a confirmed mutation carrier with a cancer diagnosis recalled the time when she suspected that, owing to her family history, she could be a mutation carrier:

They told me, “You are not a patient, you don’t have the disease. But you can get tested.” It was 4 months until I had an appointment. In that period of time, I would have wished to have somewhere to turn to, not just reading all that scary information on Google.

Female mutation carrier with cancer diagnosis, 34 years

Similarly, employees of the CIS stated that most inquiries up until this point had come from patients with cancer and their relatives (as opposed to from people without cancer). Thus, these participants concluded that trusting the live chat service would be most relevant to users for whom familial cancer had a personal relevance. These views were supported by the members of the general public who overall felt sufficiently informed by their physician or their health insurance company regarding cancer and preventive measures and therefore did not see themselves using the chat service, as described by a woman:

The web page of my health insurance company—that’s where I would go for cancer information because then I also have the information regarding the extent to which certain screening measures are covered. At the same time, I find credible information, or I am forwarded to other information portals that are also credible...In a second step, I would go directly to my doctor to get more advice.

General public, female participant, 34 years

With regard to the *sociodemographic* determinants of the trustor of a live chat, it was contested whether gender and age were relevant features of the trustor. In relation to *gender*, participants in all samples believed that women were presumably more likely to use the chat service than men. However, there was disagreement regarding whether this was a question of trustworthiness:

I think women are much more active and proactive [in the area of health]. They are also more likely to be involved in the care of patients. So I believe that women will continue to make up the larger share of users just as they do now.

Female CIS participant

However, in the focus group with only male participants, interviewees discussed the fact that Germany’s statutory cancer screening programs start as early as age 20 years for women, and therefore they receive cancer education much earlier than men, for whom these programs do not begin until age 35 years. This may result in an increased awareness among women regarding cancer prevention and exposure to cancer-related information:

There is an educational part to it [cancer knowledge]. For women in particular, as soon as they start having their periods, it’s pretty much a given that they’ll go to the gynecologist on a regular basis. But there is not the same offer for boys, for young men in their teens to have regular examinations at least once a year. That’s why I think it’s also about education and a systemic issue with the medical system.

Male mutation carrier with cancer diagnosis, 28 years

Given this lack of exposure to cancer prevention and consequently the routine habit of speaking about cancer with a physician, male participants with cancer experience deemed the anonymity of the chat service an advantage with regard to speaking to someone about their fears of suspecting cancer or questions related to their diagnosis:

What is great about this type of tool [a chat] is that it is anonymous or semianonymous. I think that’s an advantage because with men, they still often feel ashamed. They are not used to talk or don’t want to talk to someone they know. I think the anonymity could be good to talk about concerns, and you can build up trust within the first few messages.

Male mutation carrier with cancer diagnosis, 28 years

*Age* was another contested trustor characteristic: the majority of the interviewees were of the opinion that older age is related to less experience with digital tools and may thus result in lower trust in information from a live chat. However, the focus group participants described the recent COVID-19 pandemic, the implementation of digital tools for health, and many everyday life activities as responsible for reconciling age differences in health literacy and the use of digital health tools:

I would say that the type of person [using the chat] is independent of age. More important, it is the person who knows their way around the internet, who also has the confidence to find answers to health questions. It is the person who is digitally confident to operate such a live chat.

General public, female participant, 31 years

Many people aged around 80, for example, have now become very mobile phone savvy in the course of the pandemic, and they are just as capable as anyone else. So, I don’t think you can pin it down to age anymore.

Female mutation carrier with cancer diagnosis, 40 years

By contrast, *education* was viewed as a more influential factor than age. Interviewees from both the CIS and CSGs stated that, over the years, they had experienced that questions relating to familial cancer came mostly from higher educated people who were generally already well informed. This was partly explained by the complexity of the topic as well as the required skills to navigate search engines and find specialized websites such as those operated by support groups or the CIS:

It is quite often a matter of education. Hardly ever people with limited language skills or a low level of education come to us. Almost never, unfortunately. Even our website alone is too specific, how should I say, too specialized, it’s not so easy to understand.

Female CSG participant

In the view of the focus group participants, being a *member of a minority group* was another relevant feature of the trustor. Minority groups such as people with certain disabilities (eg, deafness), those with a nonheterosexual identity, or migrants were said to experience intentional and unintentional discriminatory practices in the health care sector as well as general disadvantages in finding health information targeted to their needs. In this respect, the anonymity of a live chat service could be a suitable channel for members of different minority groups:

I myself am not heterosexual, but I was always read that way. It’s put me in some really bad situations to have to put things right. For instance, when it came to mastectomy, I was the only one who said, “Maybe I will do the prophylactic surgery of the breasts without reconstruction.” And it was the same with family planning. Then you are quickly told, “Once you have a boyfriend, then you will think differently about it.” That was from the doctors and the support groups...If the people [advisers] were sensitive, I can imagine that people from the LGBTQ [lesbian, gay, bisexual, transgender, and queer] community would turn to the chat.

Female mutation carrier, 29 years

In summary, study participants identified personal experience with familial cancer and a need for further information as key determinants of a trustor for visiting the CIS website and using and eventually trusting the live chat service. Other characteristics such as being a member of a minority group, possessing higher education, and having experience with digital tools were also associated with the use of the live chat tool. Opinions varied concerning the impact of age and gender identities on tool use.

#### Relevant Determinants of the Trustee

The *humanness* of the trustee (ie, not an automated chatbot) and several subcategories for each of the theoretical constructs *ability*, *benevolence*, and *integrity* were identified as relevant trustee features in a live chat communication setting.

Participants in the focus groups repeatedly pointed out that in the era of artificial intelligence (AI), more and more commercial live chats were at least partially operated by automated chatbots. With the improvement in these bots, it has become increasingly difficult to distinguish chatbots from human respondents. In the view of participants, chatbots are suitable for superficial tasks such as returning an order from, but for fostering trust in a chat, the *humanness* of the trustee is a requisite condition:

So, I don’t think we are there yet to accept health information from a nonhuman. We talk to human doctors and when I go to the live chat and, well, if I don’t know if there’s a human sitting behind it, the question arises how well this service is going to be accepted.

General public, male participant, 30 years

Using photographs of the advisers on the CIS website or in the live chat tool as well as displaying a short biography of their professional background were measures suggested by focus group participants to show the humanness of chat advisers. In comparison, interviewees from the CIS and CSGs did not discuss the possibility of a chatbot; rather, they talked about how human cues such as visual expressions, gestures, and tone of voice could or could not be expressed and interpreted via a text-based live chat.

The subcategories of the feature *ability* were *professional (medical) expertise* and *communication skills*. Participants from all focus groups highlighted the importance of medical expertise and state-of-the-art knowledge about cancer research, prevention, treatments, and alternative therapies with regard to trusting the trustee. However, participants with a genetic mutation were critical regarding whether the personnel behind the chat service could cover the broad expertise needed to advise patients with different syndromes at diverse stages in their cancer journey and how this would affect their trust in the trustee:

If you have a very specific question about condition XYZ or treatment options...many different experts and specialists are needed. And I want my question to end up with the person who can answer it and not with another person who cannot. If I randomly connect with someone in the chat, how can I be sure of the person’s specialization?

Female mutation carrier with cancer diagnosis, 32 years

In addition, focus group participants and interviewees from CSGs also mentioned other areas of reliable professional competencies needed besides medical advice, such as help regarding social security, family, and financial support from a trusted contact person in the chat:

When I now look back, the cancer, the diagnosis was the easiest thing to deal with. And what came after that, that’s when the difficulties really started—with health insurance, household help, if you have children, and what you are entitled to get and what not. When it comes to sick pay and what happens after sick pay runs out. That’s what really sent me into free fall. I would also like to have a contact person for these types of questions.

Female mutation carrier with cancer diagnosis, 35 years

Finally, participants from all samples mentioned good communication skills as another important ability of the trustee that would further foster trust. This includes an ability to translate complex medical knowledge empathetically to inform laypeople in a manner they understand:

The chat personnel actually require a set of competencies. They have to have technical and medical authority to lead through the chat. But also some emotional competence, I think that’s what I would expect. Because if someone goes into the chat and is very upset and then only gets more technical terms or facts thrown at them, not everyone will be happy.

Female mutation carrier, 27 years

With regard to the determinant *benevolence,* our data show that *empathy* and *neutrality* are the characteristics expected from the trustee in a live chat. However, there was some variation among participants in different samples. Although participants from all samples expected empathy as a core feature of the trustee, it became apparent that participants with their own experience with the disease (eg, mutation carriers and representatives of CSGs) had the most differentiated views of how they expected empathy to be expressed. Although pity and well-meant but meaningless phrases were not desired, empathy could be shown by taking the inquirer seriously and taking time to respond to their questions individually:

Pity is what you already get in huge amounts from family and friends. What I expect from the chat person is that he or she will dedicate as much time as I need to me because that’s what doctors often don’t do. And by giving me the impression that this is an individual response to my question, that, to me, is a great expression of empathy.

Male mutation carrier with cancer diagnosis, 28 years

In addition, perceiving the trustee as neutral and unbiased further contributes to the perceived benevolence:

It should be absolutely clear that this is noncommercial without any intentions to profit. If I get the impression the service is one-sided, it would totally kill it for me.

Male mutation carrier with cancer diagnosis, 40 years

For the last trustee characteristic—*integrity*—*honesty* and *discretion* were identified as subcategories. Honesty was an often-mentioned trustee characteristic in all samples. Various participants explained that the trustee did not need to be all-knowing and should admit this:

If a question is difficult to answer, this is what should be communicated...Being honest about that, I think, that makes a live chat very credible and also very authentic.

General public, female participant, 31 years

However, advisers from the CIS had also frequently experienced that patients seeking answers could be frustrated and lose trust in the service if not provided with a clear answer:

And of course, we are always very careful, we do not make any assumptions or say something just so that the person can take something away. We are clear about our limitations. We ask them to talk to their doctor again or that the studies say this and that, but they are not quite sure yet either. But this can be frustrating.

Female CIS participant

There was repeated mention of discretion as another feature that enhances trust between trustor and trustee. Discretion was defined as treating the chat conversation between inquirer and respondent confidentially and applying high data protection standards, as the following quote illustrates:

It would be important to me that everything that is discussed in the chat is treated confidentially. Or rather, that it is communicated if a question cannot be answered and has to be passed on to a colleague.

Female mutation carrier with cancer diagnosis, 32 years

In summary, participants identified various characteristics that they associated with a trustworthy trustee in a cancer live chat service. Subcategories were identified within each of the 3 theoretical determinants: ability, benevolence, and integrity. In addition, the feature of humanness—distinguishing between human respondents and an automated chatbot—was noted as another determinant for the digital live chat.

#### Relevant Application-Related Determinants

Determinants related to the application are divided into *source* *characteristics* and *surface* *characteristics*, with several subcategories for each. Determinants relevant to the *source characteristics* revolve around the *reputation* of the institution that provides the website and the application, their *appearance*, their *user centeredness*, and the use of *state-of-the-art technology*.

The focus group participants noted the variable quality in internet information, indicating that they use reputation and institutional affiliation as initial selection and trust criteria. Many participants, including those identified as mutation carriers, were unfamiliar with the CIS, leading them to look for certified websites or quality seals as part of their trust formation process:

I go to institutions that I know, maybe the Federal Center for Health Education, because those are also trustworthy websites or I look for quality seals on websites unknown to me.

General public, female participant, 24 years

For the focus group participants, a *professional appearance* and design of the website were also factors contributing to developing trust in the CIS and using the live chat application, especially if they were unfamiliar with the service:

I would use the layout for exuding authority and trustworthiness while keeping the style and design unobtrusive.

General public, male participant, 25 years

In this service, any sign of an advertisement or a pop-up window was viewed negatively:

If I see advertisements or have to click away any windows, the whole service would suffer because I would immediately wonder whether my data would be gathered somewhere. Same with a pop-up window for the chat—if it jumps into my face, I wouldn’t perceive it as professional anymore.

Male mutation carrier with cancer diagnosis, 22 years

A *user-centered design* was highlighted by participants across all samples as the foundation to establish trust in the service overall. User centeredness encompassed barrier-free access to the website and the live chat application for people with a disability as well as chat hours that were compatible with the schedules of working adults or parents:

Many patients are working despite their diagnosis, and that’s why I think the chat should operate primarily in the evening until 8 PM, preferably until 10 PM. And weekends.

Male mutation carrier with cancer diagnosis, 51 years

Moreover, the focus group and interview participants were aware that questions often came up in quiet moments, in the evening, or even at night:

We receive many emails at night or late in the evening, and we know from some people that this is when they will start googling. So, I can imagine that it would be well received if the live chat operated late into the day.

Female CSG participant

Participants considered the use of state-of-the-art technology, particularly in the area of data protection, a prerequisite for trusting the live chat service. Specifically, mutation carriers expressed willingness to share sensitive data, such as a physician’s letter, and as a result, they expected rigorous data protection policies:

I would expect end-to-end-encryption. Or at least that I am told at the beginning of the chat that I am on an official website, and no data will be given to third parties.

Female mutation carrier with cancer diagnosis, 50 years

With regard to *surface characteristics* (ie, characteristics pertaining to the chat application’s features), participants noted the importance of the options *to remain anonymous* and *to change the medium*, as well as *fast dialogic communication*.

The flexibility to choose the amount of personal information shared and thereby the option to remain anonymous was considered a benefit of the chat:

When being diagnosed, being with all those doctors can be quite intimidating, and not everyone dares to ask all the questions they have. Also, the examinations and other stuff there [at the hospital] can be very intimate. I think the anonymity of the chat can be helpful for some people, and they might dare to ask more questions.

Female mutation carrier with cancer diagnosis, 40 years

Other participants stated that anonymity could hinder establishing trust in the person they were chatting with, and they suggested that a video tool should be implemented in the program:

A clear disadvantage to the anonymity of the live chat is that I don’t have that emotional component. I don’t know if anyone can take away my insecurity via a written live chat. If I could turn on a video and know I am talking to a real person—I personally would trust the video chat more.

General public, male participant, 28 years

Implementing a *change of medium* either to video or telephone was also suggested to address participants’ concerns that familial cancer covered a wide range of complex topics that could not be adequately discussed in a chat window:

With hereditary cancer, you will probably find yourself relatively quickly at a level when you have to ask complex and existential questions. I don’t think these are easy to deal with in a chat like this, and you will need to have a good strategy. How do I refer them to any of our own services? How do I transfer them to somewhere else where they can be helped?

Female CIS participant

Finally, participants described live chats as *fast dialogic communication* and anticipated that the planned live chat would facilitate a consistent, although not necessarily instantaneous, conversation with their chat partner:

The advantage in contrast to counseling via email is that you simply get much faster feedback and in smaller bites that are easier to consume...I don’t want to define a cutoff time when I am no longer willing to wait for a response, but I think the medium lives by its quickness.

General public, female participant, 34 years

Participants indicated willingness to wait for responses, although the acceptable waiting times varied among them. Long pauses and uncertainty about the trustee’s activities during these pauses were identified as factors that could affect trust perceptions:

I at least want to know they are busy researching an answer, and I am willing to wait. If I don’t hear from them for more than 5 minutes, not even saying “I am still with you, please wait,” it would make me nervous.

Female mutation carrier with cancer diagnosis, 46 years

In summary, study participants identified a range of factors for application-related source characteristics and surface characteristics as essential for using the live chat. These factors include the reputation of the institution as well as the appearance, user centeredness, and state-of-the-art technology of the chat application itself.

### Comparison of Samples and Sociodemographic Differences Regarding Trust Determinants (RQ2)

This section compares overlaps and differences among the samples as well as sociodemographic data in relation to the 3 dimensions under research. Comparative sample citations from each sample for each determinant in conformity with Figure 1 are summarized in Table 4. As this is a qualitative sample, it should be mentioned at this point that no systematic comparison can be made as in the context of quantitative samples.

The highest level of agreement across all samples was observed in the trustee determinants for a cancer-related live chat. Participants identified the humanness of the trustee as a key factor for trust. They suggested that providing information about the CIS live chat advisers could accentuate their humanness, differentiating them from chatbot-operated chats.

Although there was general consensus regarding the trustee’s expected characteristics of ability, benevolence, and integrity, there were slight variations in how these characteristics should be manifested; for example, in German, there is a difference between formal and informal modes of address. Some participants, particularly those working at the CIS, preferred the formal mode of address because it maintained professional distance while demonstrating empathy. This view was shared by approximately half of the focus group participants, whereas others believed that the formal mode of address could create an unwarranted distance:

I am addressing the user not as a friend but as an expert or doctor with an institutional background. I thus have to keep a professional distance, and the formal mode of address doesn’t interfere with my empathy for them.

Female CIS participant

If I was addressed with the formal mode of address, I would not feel a connection, and it would prevent me from opening up.

Male mutation carrier, 28 years

Our study indicates that trustee determinants are closely linked with application-related determinants, suggesting that trust formation toward the trustee begins even before the trustor engages personally in the chat through the assessment of the institution, its website, and application features. In addition, the trustor’s perceptions of the trustee in the chat are influenced by their overall experience of the CIS website and their trust in the institution. Younger participants from the focus group discussions, many of whom belong to the digital native generation, mentioned more specific requirements to enhance the trustworthiness of the website and application compared with the overall older interview participants. Thus, although professionalism and thematic authority were often-mentioned application-related determinants of trust in all samples, younger digitally experienced participants had detailed requirements regarding how these characteristics should be reflected in the design of the website and live chat application.

A suspicion or diagnosis of familial cancer (hence, a specific health status) was viewed as an essential trust-relevant characteristic, along with digital skills, and prior experience with live chats. However, whether these skills were age specific and increasingly found among younger trustors was contested in our study, with differing views between the interview and focus group participants. Sociodemographic characteristics (eg, age, gender, or education of the focus group participants, ie, the potential users) did not indicate strikingly different answers in our analysis.

In summary, despite the vastly different backgrounds and sociodemographic data across our samples, many overlaps were found for all 3 determinants under research. The greatest differentiations were found in the description of details relating to the determinants of the trustee and the application (source determinants and surface determinants); in this respect, the focus group participants who were recruited for their perspectives as potential users stood out.

**Table 4:** Comparison of trust determinants across the 3 distinct samples.

Dimensions and determinants		Cancer Information Service employees	Cancer support group representatives	Potential users
**Trustor**				
	Health status (personal cancer experience)	“Most often it’s actually patients and family members, so half and half, patients and family members who contact us. While we now have more older and middle-aged inquirers, we want to appeal to a younger audience with the chat.” (Female participant)	“From our experience, it’s mostly women who are already well informed about gene mutations, even if they are not affected, but a relative...For the chat, I would expect a younger audience.” (Female participant)	“They told me, ‘You are not a patient, you don’t have the disease. But you can get tested.’ It was 4 months until I had an appointment. In that period of time, I would have wished to have somewhere to turn to, not just reading all that scary information on Google.” (Female mutation carrier with cancer diagnosis, 34 years)
	Sociodemographic features (age)	“I think that especially the younger generation will be the users, they don’t like to talk on the phone that much anymore.” (Female participant)	“Of course, they [younger generations] deal with it in a completely different way, but even the older people would get their grandchildren or children and somehow get the technology to work. So I don’t think these hurdles are as big as they appear.” (Male participant)	“I would say that the type of person [to use the chat] is independent of age...It is the person who is digitally confident to operate such a live chat.” (General public, female participant, 31 years)
**Trustee**				
	Humanness	“What’s problematic is that there are humans on both sides of the chat, but many of the cues that make us humans are missing. I can’t see if the person understands what I write or not.” (Female participant)	“I would say it’s a bit more impersonal. Through the chat, you still have a certain distance because you can hide behind your computer. In a phone call, well, you can also understand certain nuances in the voice or even the urgency of the person concerned, which you can of course make much clearer in a conversation. Yes, that is definitely a challenge.” (Male participant)	“So, I don’t think we are there yet to accept health information from a nonhuman...If I don’t know if there’s a human sitting behind it, the question arises how well this service is going to be accepted.” (General public, male participant, 30 years)
	Ability (professional medical expertise)	“Of course, it is very important that our work is evidence based and reliable. We have a very large team that always researches all cancer topics in detail, is well trained, so they are all experts who know what they are doing.” (Female participant)	“We should be transparent about the boundaries of our knowledge. We as support groups have different knowledge than the CIS [Cancer Information Service] and acknowledging that and making referrals is also something that I consider important for the operation of the live chat.” (Female participant)	“And that’s why it immediately occurred to me, when you think about designing the chat in some way, that you feel a bit more trusting there. That you can at least see a profile picture or a name: ‘Is this a professional, is this a doctor I’m chatting with?’ or something like that.” (Male mutation carrier with cancer diagnosis, 27 years)
	Benevolence (empathy)	“In a chat, you can actually get a feeling for the person on the other side and that makes it easier to show empathy. That’s a difference compared with our email service where we have almost no interaction, and I find it difficult to be more personal, although I think it’s important.” (Female participant)	“I think being empathetic is key because it makes it easier to ask my questions, especially the questions that really bother me deep down. But it might also be a challenge in the live chat because with writing, there is potential for misunderstandings.” (Female participant)	“I expect professional competence in a person who is supposed to lead such a chat. But also emotional competence. I think the person needs both.” (Female mutation carrier, 27 years)
	Integrity (discretion)	“But of course you have to make sure that no information is passed on to third parties and that nothing is stored. So I think communicating this information will probably be important to the people.” (Female participant)	“Data protection is a big topic, especially in the area of hereditary cancer when you have to ask some questions about the family history of the person in order to give them relevant advice...So allowing for a certain anonymity and also sharing personal medical data need to be thought about beforehand.” (Female participant)	“Yes, of course it would be important to me that everything that is discussed in the chat is treated confidentially. Or rather, that it is communicated if a question cannot be answered and has to be passed on to a colleague, that this is then communicated. Yes, and that everything else remains, I’ll say, in private, which is not said again separately.” (Female mutation carrier with cancer diagnosis, 32 years)
**Application**				
	Source characteristics	“I think the technology, that’s actually something that’s very disruptive when it doesn’t work. And it’s annoying in all areas, but I think it’s especially bad in chat, yes. It should run reliably.” (Female participant)	“You would have to, in my opinion, have a live chat that is relatively barrier free through the website for this to work” (Male participant)	“I remember from my chemotherapy that I kept forgetting things and I would therefore like it if I could save or print the chat for later review...What I am saying is that the technological features should suit the needs of the users, and you should think about that too.” (Female mutation carrier with cancer diagnosis, 34 years)
	Surface characteristics	“Maybe they [chat users] even reveal more about themselves or dare to say something or ask something that they wouldn’t otherwise ask because they perhaps have the feeling that they are even more anonymous. Just by not showing a phone number or an email address. Perhaps they will then also dare to say something more.” (Female participant)	“Because there are also topics, I personally don’t find them so delicate, but which are perhaps still seen as such in society. Partnership, sexuality, all such things, where it might help if you had the possibility to choose a pseudonym, that is, in the chat. That would definitely be a good option.” (Male participant)	“So I think the live chat will fill a gap, especially for people who have an increased barrier to talk about the issue.” (General public, male participant, 30 years)

## Discussion

### Principal Findings

Trust is of great importance in situations marked by risk and uncertainty and, thus, essential to health information seeking [9,70]. The digital transformation has altered how users consume and interact with health information. Consequently, understanding trust formation for new technologies such as live chats is more important than ever. Moreover, in the field of familial cancer risk, its complexities and sensitivities combined with the vulnerability of those seeking information about this topic underscore the urgency of examining trust determinants for this new service. It is crucial to understand how the determinants of trust interplay and shape trust formation to ensure that the service meets the needs of its users and supports them in dealing with their concerns and questions. Thus, we conducted a study to gain a better understanding of the determinants of trust for a live chat service for medical information.

On the basis of a triangular concept, we focused on the determinants of trust on 3 distinct dimensions (trustor, trustee, and application; Figure 1). Our data indicate that the general determinants of the trust dimensions are associated with other communication formats (eg, static website content), but certain determinants are specific to live chats, which will be the focal points of this discussion.

Regarding the *trustor*, personal experience with familial cancer, combined with a need for additional information, emerged as a key determinant to seek, use, and eventually place trust in the live chat service offered by the CIS. These determinants suggest that the live chat service is of particular relevance to those dealing with familial cancer or familial cancer risk, highlighting the value of offering such a personalized and accessible medium for information exchange.

Other characteristics, including being part of a minority group and experiencing unmet needs in traditional face-to-face health care (eg, the lesbian, gay, bisexual, transgender, queer, intersex, asexual, and similar minority [LGBTQIA+] community) and possessing prior experience with digital tools, were also identified as factors influencing the use of, and trust formation toward, the live chat service. Our study also indicated varying opinions regarding the impact of age and gender identities on the use of the live chat service. This points toward the complexities inherent in trust formation and use patterns, necessitating further investigation to better tailor the service to diverse user needs and preferences. It also implies that trustors’ decisions to engage with, and trust, the live chat service are multifaceted, informed not only by their personal experiences and needs but also by their individual characteristics and backgrounds. Thus, acknowledging and addressing these diverse factors in the design and promotion of live chat services could help to enhance their reach and effectiveness.

When characterizing the *trustee*, participants highlighted a range of factors that were primarily categorized under the 3 theoretical determinants of trust—ability, benevolence, and integrity—with each offering unique insights into the formation of trust in this digital service.

When considering the *ability* aspect, it becomes apparent that the expertise and qualifications of the trustee play a pivotal role in establishing trust. This implies that reinforcing the credentials and qualifications of the human advisers in the live chat service could enhance the trustor’s trust in the information provided. *Benevolence*, or the perception that the trustee acts in the interest of the trustor, suggests that the manner in which the live chat service is delivered—emphasizing empathy, understanding, and an interest in the questions of the trustor—can influence the trust formation in the trustee. Similarly, *integrity*, or the belief in the honesty and moral principles of the trustee, emerged as an important factor. This highlights the importance of transparency and data protection in interactions within the live chat service to build and maintain user trust. Moreover, our study also found that the *humanness* of the live chat service—whether the responses were generated by a human or an automated chatbot—was a significant determinant of trust. This underlines the value that users place on human interaction, particularly in the sensitive context of discussing cancer. In the rapidly evolving landscape of AI technology and its potential implications for future health-related chat services, managing user expectations and perceptions of *humanness* require careful consideration. Although our research focuses on a live chat service operated exclusively by human medical advisers, we recognize that advancements in AI could reshape user perceptions and trust formation processes in the future. Our study findings underscore the importance of the human element in fostering trust within a chat service for users with a high need for certainty. For a chat operated by humans, this means implementing cues that showcase their expertise as relevant (eg, by integrating biographies and possibly photographs of the advisers on the CIS website to differentiate the CIS live chat from other chatbot-operated chats). For AI-supported chats, it would be important to inform users transparently about the inclusion of AI backed by human expertise to nurture trust. As AI continues to evolve, future studies might also examine how to optimally blend AI efficiency with the empathy and understanding that a human adviser brings to ensure user comfort and trust.

With regard to *application-related features*, several key factors were identified that may affect the decision to use and trust the live chat service, revolving around both source characteristics and surface characteristics. Notably, these included the institution’s reputation, the chat application’s appearance, user-centered design, and the use of state-of-the-art technology. It is worth noting that the assessment of the source determinants and surface determinants of trust typically occurs in a sequential process. Initially, prospective users of the live chat service form a preliminary impression of the trustee’s authority and expertise based on the overall website presentation and content. This initial impression seems to influence the decisions to engage with, and use, the live chat service.

This sequential process of trust formation underscores the necessity of an intuitive user-friendly design that showcases the organization’s credibility and expertise. Source determinants of the application might also be built in offline environments if the organization, brand, or institution—in this case, the CIS—behind a website is already known and deemed trustworthy [4,71,72]. Moreover, we acknowledge that the decision to use and trust a live chat service might be entangled with various factors that extend beyond the immediate interaction (eg, its endorsement by another trusted organization or referral source or the service’s ranking in search engines) [43].

To investigate the determinants of trust, we drew on the perspective of 3 different samples—CIS employees, volunteers from CSGs, and potential users with experience with hereditary genetic mutations as well as those without—through interviews and focus group discussions. Despite the professional and personal differences among the participants in these samples, we found many overlaps and complementing views concerning the determinants of trust under investigation. This multiperspective study design allowed us to understand trust as a dynamic interplay of trustee, trustor, and application-related determinants [4]. Moreover, trust in digital health interventions, such as a live chat service, evolves not only during the user experience but also beyond it. Factors such as a quality seal on the website or recommendations can influence initial trust. During the interaction, elements such as empathetic communication and the personal mode of address of the user serve to build this trust.

### Limitations and Future Research

First, owing to the qualitative nature of our study, we could not test the relationships between the dimensions and their respective determinants of trust. Moreover, our main recruitment criterion for focus group participants was their experience with familial cancer (ie, no or known genetic disposition to cancer with cancer experience or without). Hence, we can draw limited conclusions about other sociodemographic characteristics (ie, age and educational level) and their relation to trust formation. This means that the dynamics as well as the importance of certain subcategories and sociodemographics of participants need to be tested in a quantitative study with a larger sample.

Second, in our study, we aimed to explore the 3 determinants separately to better understand them; yet, these elements are intertwined and collectively contribute to the overall trust formation process. Moreover, trust-influencing factors may go beyond what we focused on in our study; for instance, a user-friendly interface, the website domain, and its ranking on search engines can enhance perceived trustworthiness by creating a positive user experience, thereby indirectly fostering trust. Given this interconnectivity, further investigation is required to fully understand how these elements interact and influence one another in the context of trust formation.

Third, the CIS was not well known among the potential users of the live chat service, whereas it was well known among the interviewees (CIS employees and representatives of CSGs). Therefore, an exploration of institutional reputation on trust formation among potential users could not be carried out.

Fourth, a self-selection bias is likely in our sample, especially among the sample of potential users. The higher proportion of women compared with men in our study reflects the general gender ratio of cancer information seeking; for example, a long-term analysis of requests to the CIS has shown that inquiries by women have always made up the majority [73,74]. Moreover, all focus group participants seemed to be familiar with digital devices and navigating the internet; hence, a study to explore the needs of groups with less digital health literacy might be worthwhile.

Fifth and last, in our study, the terms *trust* and *use* were closely related, although they should be methodologically and analytically differentiated in future research [15].

### Conclusions

Trust is a well-developed field of inquiry in research, and there is agreement that because it is multidimensional and highly context dependent, the determinants of trust must be explored in their specific context and, in web-based environments, in relation to their application. Although web-based live chats are regularly used for commercial purposes, their application for personalized health information and advice regarding complex diseases such as familial cancer remains underexplored. By triangulating 3 different perspectives in a qualitative study, we have created an in-depth understanding of the interplay among the multiple facets of the 3 dimensions of trust and their determinants for a live chat at the German CIS. Concerning the determinants of the trustor, that is, the person who trusts, we noted that having a personal experience with a disease is key to using and potentially trusting the live chat service. The live chat might also be particularly valuable for people from minority groups (eg, the LGBTQIA+ community) who experience discrimination in physician-patient interactions. We further identified an array of features that determine trust in the trustee, that is, the person to be trusted, and the application through which the interaction is mediated. We concluded that in the era of chatbots, having human advisers is a necessary requirement for trustors to use the chat and share information regarding their diagnosis or concerns. We thus recommend highlighting this information before the chat entry point, that is, on the institution’s website, via biographies and photographs. Finally, although we could establish the existence of a dynamic triangular interplay among the different determinants of trust, we encourage researchers to investigate these relationships with a model in a quantitative study.

## Abbreviations

AI: artificial intelligence
CIS: Cancer Information Service
CSG: cancer support group
LGBTQIA+: lesbian, gay, bisexual, transgender, queer, intersex, asexual, and similar minority
RQ: research question
